# Validated Quantification of HHV-8 DNA Using Inter-Convertible Plasmid and Cell-Derived Calibrators: Optimization of a Whole-Blood qPCR Assay

**DOI:** 10.3390/v18050578

**Published:** 2026-05-21

**Authors:** Celeste Luján Pérez, Carlos Ochoa Gamboa, Mónica Tous, Julián Hazan, Marcelo Rodríguez, Daniela Feliciotti, Lucía Irazu, Carlos Zala

**Affiliations:** 1 Tissue Culture Service, Virology Department, INEI-ANLIS “Dr. Carlos G. Malbrán” (1282AAF), Buenos Aires C1282AAF, Argentina; carlos8ag@gmail.com (C.O.G.); mtous@anlis.gob.ar (M.T.); jhazan@anlis.gob.ar (J.H.); dfeliciotti@anlis.gob.ar (D.F.); 2 INEI-ANLIS “Dr. Carlos G. Malbrán”(1282AAF), Buenos Aires C1282AFF, Argentina; marcerodriguez2002@gmail.com (M.R.); lucirazu@anlis.gob.ar (L.I.); 3 Department of Microbiology, School of Medicine, University of Buenos Aires, Buenos Aires C1121A6B, Argentina; zalacarlos@gmail.com

**Keywords:** HHV-8, KSHV, real-time PCR, viral load, Kaposi’s sarcoma, whole blood, ORF26

## Abstract

Human herpesvirus 8 (HHV-8) is the etiologic agent of Kaposi’s sarcoma (KS), primary effusion lymphoma (PEL), multicentric Castleman disease (MCD), and KS-associated immune reconstitution inflammatory syndrome (IRIS-KS). Quantifying HHV-8 DNA in whole blood is clinically relevant, yet laboratory practices remain heterogeneous. Here, we developed and validated an in-house quantitative PCR (qPCR) assay targeting ORF26, optimized for whole blood. Assay calibration used plasmid, BCBL-1 cell–derived, and commercial HHV-8 DNA standards. Analytical validation was performed following the Clinical and Laboratory Standards Institute (CLSI) guidelines and the Minimum Information for Publication of Quantitative Real-Time PCR Experiments (MIQE) guidelines and showed a 95% limit of detection of 65.7 copies/reaction, efficiencies of 90–101% (R^2^ > 0.99), and intra/inter-assay coefficients of variation < 6.5%. Strong correlations were observed among the three calibrators (R^2^ > 0.97).Clinical validation against a composite reference yielded 100% sensitivity, specificity, PPV, and NPV. Viral loads (log_10_ copies/mL) varied by clinical condition: classic KS and transplant-associated KS showed the lowest medians (2.30–2.23), MCD HIV− and PEL intermediate values (2.83–3.72), and epidemic KS, MCD HIV+, and IRIS-KS the highest (4.12, 4.86, and 5.03, respectively). Viremia > 5 log_10_ copies/mL was associated with uncontrolled E-KS, MCD HIV+, and IRIS-KS. Longitudinal follow-up revealed viral load decline paralleled clinical improvement. This validated assay provides a robust, affordable tool for HHV-8 quantification in whole blood and supports its integration into diagnostic workflows and patient monitoring.

## 1. Introduction

*Human gammaherpesvirus 8* (HHV-8), classified within the family Herpesviridae, subfamily Gammaherpesvirinae, and genus Rhadinovirus, is also known as Kaposi’s sarcoma-associated herpesvirus (KSHV). It is the etiologic agent of Kaposi’s sarcoma (KS), primary effusion lymphoma (PEL), multicentric Castleman’s disease (MCD), and acute episodes such as Immune Reconstitution Inflammatory Syndrome Associated Kaposi Sarcoma (KS-IRIS) or Kaposi Induced Cytokine Syndrome (KICS) [1,2].

Quantification of HHV-8 DNA in whole blood by real-time polymerase chain reaction (qPCR) is a key tool for diagnosis, disease monitoring, and therapeutic evaluation in patients with HHV-8-associated conditions. Several studies have underscored the importance of viral load (VL) as a biomarker for disease burden and progression risk, particularly in immunocompromised individuals [3].

Despite its clinical relevance, the lack of standardized protocols and the heterogeneity in methodologies across laboratories highlight the need for robust, reproducible, and clinically validated qPCR assays [4]. Commercial tests are not always available or suitable, especially in resource-limited settings. In this context, in-house qPCR platforms provide a cost-effective and adaptable alternative for routine diagnostics and patient management.

Quantitative detection of HHV-8 DNA in whole blood has been shown to provide high sensitivity and strong clinical correlation, as this matrix reflects both cell-associated and circulating viral DNA [5,6]. However, significant heterogeneity persists in how viral load is reported across studies—whether as copies per milliliter of blood or plasma, per nanogram of DNA, or per 10^6^ cells—making direct comparison between laboratories difficult and hindering the establishment of reference thresholds for clinical interpretation [3,7,8].

Several genomic regions have been explored for HHV-8 detection, most notably ORF26, ORF73 (LANA), and K6, with ORF26 demonstrating optimal analytical stability and genotype coverage.

Speicher and colleagues first described quantitative PCR assays targeting ORF26 and ORF73 for HHV-8 DNA detection [7], establishing ORF26 as a robust and diagnostically reliable target that has since been adopted in several research and clinical laboratories. The clinical utility of ORF26-based quantification has also been demonstrated using commercial assays in patients with KSHV-associated inflammatory syndromes [8], while assays directed to K6 have been applied to evaluate viral IL-6 expression and systemic inflammation in multicentric Castleman disease [6]. Importantly, ORF26 is also employed in CE-IVD commercial systems such as the HHV-8 ELITe MGB^®^ Kit (ElitechGroup, Puteaux, France), the GeneProof HHV-8 PCR Kit (GeneProof a.s., Brno, Czech Republic), and the Quanti HHV-8 PCR Kit (Clonit S.r.l., Milan, Italy), underscoring the clinical relevance and comparability of this target.

Nevertheless, despite these advances, a unified calibration and reporting framework is still lacking, which limits reproducibility and the clinical transferability of quantitative results. Building upon this evidence, our study applies a validated calibration framework to whole-blood quantification, enabling reproducible conversion between plasmid, cell-derived, and commercial standards for HHV-8 viral-load measurements.

The HHV-8 ORF26 fragment has been employed in our laboratory for viral detection in clinical samples and to study the molecular epidemiology from our region [9,10,11]. This report outlines technical and analytical strategies to establish a qPCR assay targeting the ORF26 region, optimized for whole blood. The protocol incorporates plasmid and cell-derived calibrators and includes comparative evaluation with commercial control.

The aim of this study was to develop and validate an in-house qPCR assay for quantifying HHV-8 DNA in whole blood. Analytical performance—including sensitivity, specificity, linearity, efficiency, and reproducibility—was evaluated using plasmid, cell-derived, and commercial calibrators. The correlation among calibrators and the assay’s clinical utility was assessed in a cohort of patients with confirmed KSHV-associated diseases.

## 2. Materials and Methods

### 2.1. Primers and Probes Design

Two TaqMan probe systems were evaluated: a FAM–TAMRA probe and a FAM–MGB non-fluorescent quencher (NFQ) probe. The FAM–MGB system was designed using Primer Express™ v3.0.1, whereas the FAM–TAMRA system was generated using the PrimerQuest™ Tool ((Integrated DNA Technologies, Coralville, IA, USA; accessed January 2019).

Primer design was based on a multiple alignment that included all available reference sequences for ORF26 genotypes (A/C, J, D, B, K, and R) together with local sequences previously characterized in our laboratory (GenBank accessions KU987585–KU987608) [12] (Figure S1). Site-directed variants were incorporated as detailed in Table S1.

### 2.2. Cell Lines

Three cell lines from the Tissue Culture Biobank of ANLIS-INEI “Dr. C. G. Malbrán” were used: BCBL-1 (KSHV^+^/EBV^−^), obtained through the NIH AIDS Reagent Program (USA), for calibrator preparation; Ramos (KSHV^−^/EBV^−^) as the negative control; and P3HR1 (KSHV^−^/EBV^+^) to assess assay specificity.

All cell lines were maintained under standard culture conditions, routinely screened for mycoplasma, and authenticated by short tandem repeat (STR) profiling. Cryopreserved stocks were maintained in liquid nitrogen.

### 2.3. Calibrators and Controls

#### 2.3.1. Cell-Derived Calibrator

HHV-8 DNA was extracted from BCBL-1 Lot 220601 (7 × 10^6^ cells). After thawing, and centrifugation, the pellet was adjusted to 700 µL. Seven 100 µL extractions were performed using the proteinase K protocol, and resuspended in Tris-EDTA (TE) buffer. Extracts were pooled, quantified, and viral copies/µL estimated assuming 6.6 pg DNA/cell and 80 viral genomes/cell [12].

Serial tenfold dilutions from 10^6^ to 1 copy/reaction were prepared from a stock of ~2 × 10^6^ copies/µL. This calibrator served as primary standard to evaluate reproducibility and equivalence with plasmid-based material.

#### 2.3.2. Plasmid Calibrator

The pKS330 BAMHI plasmid (AIDS Reagent Program, NIH, USA) containing a 330 bp ORF26 insert cloned into pCRII was propagated in *E. coli* Top10 on LB–ampicillin plates. Following colony PCR confirmation, plasmid DNA was purified and quantified.

Copy number was estimated using the online “Calculator for Determining the Number of Copies in a Template”, yielding 6.35 × 10^9^ copies/µL in 82 µL [9]. Tenfold dilutions from 10^6^ to 1 copy/reaction were prepared in TE pH 8.0.

#### 2.3.3. Commercial Control

A purified HHV-8 DNA control (AMPLIRUN^®^ HHV-8 DNA, Vircell MBC128-R, Granada, Spain; 16,000 copies/µL) was analyzed in parallel with the BCBL-1 calibrator for the range 10^5^–10^2^ copies/reaction.

#### 2.3.4. Correlation and Batch Variability

Correlation between calibrators was assessed using standard curves generated from plasmid DNA and BCBL-1–derived extract (Lot 220601), using tenfold serial dilutions (10^6^–10^1^ copies/reaction), tested in triplicate across five consecutive days. For each dilution point, descriptive statistics were calculated (mean Ct, SD, 95% CI, median and IQR). Amplification performance for each calibrator was examined by plotting Ct values against the logarithm of input copies and fitting simple linear regression models [13]. These analyses were used to determine whether a quantitative relationship could be established between plasmid-based and cell-derived standards.

Because the commercial HHV-8 DNA control was available only in limited volume, calibration curves for this material were generated within the 10^5^–10^2^ copies/reaction range. Linear regression was applied to compare its amplification behavior with that of the BCBL-1–derived calibrator under matched experimental conditions. In addition, non-parametric comparisons (Mann–Whitney U test) were performed at corresponding dilution points to document similarities or differences in Ct distributions.

To evaluate potential batch-to-batch variability within the cell-derived calibrator, additional standard curves were prepared from BCBL-1 Lots 050809 and 150111 using the same dilution series. Comparisons among lots were performed by assessing regression parameters (slope and R^2^), and analysis of covariance (ANCOVA) was applied to determine whether slopes or intercepts differed significantly among batches.

This methodological framework enabled the determination of whether calibration factors or conversion relationships were required when using plasmid-derived versus cell-derived materials, and whether variability among independent BCBL-1 batches needed to be accounted for in subsequent analyses.

### 2.4. Viral Controls for Specificity

Viral stocks of HHV1, HHV2, VZV were provided from Neurovirus Service and CMV from Congenital & Perinatal Virus Service, at the Virology Department, INEI- ANLIS “Dr Carlos G Malbrán” (1282AAF) Buenos Aires, Argentina; EBV was obtained from P3HR1 cell line.

### 2.5. External PCR Inhibition Control (β-Globin and GAPDH)

To address potential concerns regarding PCR inhibition in clinical samples, β-globin amplification was performed as part of the routine endpoint PCR workflow [10] in all samples. GAPDH real time amplification [14] was performed in a separate reaction to confirm successful amplification. These assays served as external controls to confirm adequate DNA quality and the absence of inhibitory substances in whole-blood extracts.

### 2.6. qPCR Assay Optimization

Initial optimization was performed using 10^3^ copies of DNA extracted from BCBL-1 cells. A temperature gradient (60 °C, 58 °C, 57 °C, 56 °C, and 52 °C) and a range of primer and probe concentrations were tested according to the Applied Biosystems Absolute Quantification Getting Started Guide (PN 4304449), which recommends primers between 50–900 nM and probes between 50–250 nM.

Subsequent evaluations included calibration curves at 10^2^–10^4^ copies to compare detection systems (FAM–TAMRA vs. FAM–MGB). The two best-performing primer–probe sets were selected based on ΔRn, Ct values, and SD. Full six-point calibration curves (10^6^–10^1^ copies) were then generated to compare efficiencies, allowing selection of the optimal detection chemistry.

### 2.7. Analytical Validation of the qPCR Assay

Analytical validation was performed using the BCBL-1 cell-derived calibrator as the working standard, following CLSI EP05-A2 [15], EP6-A [16] and the MIQE guidelines.

Tenfold serial dilutions (10^6^–10^1^ copies/reaction) were analyzed by two operators over 20 non-consecutive days, with replicate measurements at each dilution point.

Linearity was assessed by fitting regression curves of Ct vs. log_10_ (copies). Amplification efficiency was calculated using E (%) = (10^–1/slope^ − 1) × 100. Performance was summarized by slope, intercept, and R^2^.

Precision was evaluated from mean Ct, SD, and CV% across all points. Inter-operator agreement was examined by comparing slopes and R^2^ values and by summarizing Ct variability (CV%) across days.

The reportable range was established from the precision study as the limits of the linear range. Results within this interval were multiplied by the extraction correction factor to express viral load either as log_10_ copies/mL of whole blood or as log_10_ copies/ng of DNA.

The limit of detection (LOD) was subsequently estimated by the Probability of Detection (PODLOD) approach [14].

Clinical precision was assessed by testing two whole-blood samples containing approximately 10^4^ (high control) and 10^2^ (low control) copies/reaction over 10 non-consecutive days (four replicates/day), enabling estimation of repeatability, intermediate precision, and intralaboratory precision.

#### 2.7.1. Evaluation of Matrix Effect on Calibration Curves

To examine matrix-related effects on amplification, calibration curves were generated using the BCBL-1 standard diluted either in TE buffer or spiked into a constant background of 150 ng of DNA extracted from HHV-8–negative whole blood. Four concentration points within the validated linear range (10^5^, 10^4^, 10^3^, and 10^2^ copies/reaction) were tested in triplicate. The routinely used BCBL-1–derived calibrator was included in each experiment for comparison. Non-template controls (TE-only and matrix-only) were included to verify the absence of contamination or background amplification.

The analytical comparison focused on evaluating slope equivalence and amplification efficiency across BCBL-1 (TE) and BCBL-1 + matrix.

#### 2.7.2. Clinical Validation (Qualitative Assessment)

To evaluate diagnostic concordance in blood samples, the in-house qPCR assay and a conventional nested PCR targeting the ORF26 region -routinely used for HHV-8 diagnosis [10,11] were compared against a composite clinical classification [17].

The clinically positive group (*n* = 55) included individuals with established HHV-8–associated diseases (KS, MCD, PEL) and fulfilled at least one of the following:

(i) HHV-8 DNA detected by nested PCR in blood, saliva, lymphocytes, or tissue biopsy;
(ii) anti–HHV-8 IgG seropositivity.

The clinically negative group (*n* = 66) consisted of patients referred for differential diagnosis of conditions unrelated to HHV-8 infection, all seronegative and consistently negative by nested PCR.

DNA was extracted from 200 μL of whole blood using the QIAamp DNA Mini Kit (Qiagen GmbH, Hilden, Germany), eluted in 50 μL of elution buffer and quantified with a NanoDrop spectrophotometer. Identical DNA amounts (150–200 ng) were used for both nested and qPCR, in accordance with the quantities established for the routine nested PCR diagnostic protocol.

Contingency tables were constructed for each method against the clinical classification. Diagnostic sensitivity, specificity, PPV, and NPV were calculated. Direct comparison between qPCR and endpoint PCR was performed using McNemar’s test for paired samples (*p* < 0.05 considered significant).

### 2.8. Blood Viral Load (VL) Analysis from HHV8 Associated Diseases

#### 2.8.1. Viral Load Expressed as log_10_ Copies/mL of Whole Blood

Beyond assay validation, HHV-8 viral loads expressed as log_10_ copies/mL were analyzed in 35 patients (7 women, 28 men; age 19–86 years) with confirmed HHV-8 infection based on endpoint PCR [10,11] and anti–HHV-8 IgG serology [11,18]. Diagnosis categories included: classic KS (C-KS, *n* = 4); epidemic KS (E-KS, *n* = 15); IRIS-associated KS (IRIS-KS, *n* = 4); MCD in HIV-positive (MCD HIV+, *n* = 7); and in HIV-negative individuals (MCD HIV−, *n* = 2); PEL, *n* = 1; post-transplant KS (TX-KS, *n* = 2). A summary of baseline samples included in the viral load analysis (*n* = 35), longitudinal follow-up samples (*n* = 9), and clinically positive cases without sufficient residual material for VL testing (*n* = 11) is provided in Table S8 clarifying the relationship between the diagnostic cohort evaluated in Section 3.4 and the subset available for quantitative analysis.

DNA was extracted from 200 µL of whole blood using the QIAamp DNA Mini Kit (Qiagen) and eluted in 50 µL of elution buffer. For qPCR, 5 µL of eluted DNA was used per reaction. Viral loads were calculated using:

$$copies/mL=copies/reaction\times \frac{50\;µL}{0.2\;mL\times 5\;µL}$$

Descriptive statistics summarized VL distribution per clinical group. Global comparisons used the Kruskal–Wallis test, and pairwise comparisons were evaluated using Mann–Whitney U tests with Bonferroni correction.

#### 2.8.2. Viral Load Normalization by DNA Content (log_10_ Copies/ng DNA)

To assess the effect of normalization on intergroup comparisons, viral loads were also expressed as log_10_ copies per ng of extracted DNA. Viral load (VL) values expressed as log_10_ copies/ng DNA and as log_10_ copies/mL of whole blood were analyzed sequentially. Clinical groups were first plotted to visualize the distribution and median VL within each normalization unit, and differences among medians were examined to determine whether overall group rankings were consistent across normalization scales. This alternative normalization was applied to all clinical samples included in the viral load analysis (2.8.1) to evaluate whether expression per unit of DNA produced comparable distribution patterns across clinical conditions.

## 3. Results

### 3.1. Assay Optimization

The most stringent and sensitive performance was observed at 52 °C. Final cycling conditions included three stages: 50 °C for 2 min, 95 °C for 10 min, followed by 40 cycles of 95 °C for 15 s and 52 °C for 1 min.

Primer and probe combinations was tested using 10^3^ copies per reaction for both FAM-TAMRA and FAM-MGB systems. Two sets per system with the lowest Ct and SD values were selected. The full dataset is available in Table S2. For FAM-TAMRA, combination A (250–300/900 nM) had a Ct of 30.2 ± 0.028, and combination B (125–300/900 nM) had 31.3 ± 0.15. For FAM-MGB, combination A (125–900/300 nM) showed 31.09 ± 0.014, and combination B (125–900/900 nM) had 30.0 ± 0.014.

Efficiency and linearity were assessed via three-point calibration curves (Figure S2). All combinations showed strong correlation coefficients (R^2^ ≥ 0.99). FAM-TAMRA A achieved a slope of 4.35 (R^2^ = 0.9926), and FAM-MGB A, a slope of 3.6 (R^2^ = 1).

These combinations were then compared in a six-point calibration curve (10^6^ to 10 copies). Both systems showed linear amplification over the entire range (R^2^ = 0.998). FAM-MGB consistently detected as low as 10 copies per reaction, while FAM-TAMRA was reliable from 10^2^ copies. Table S3 provides detailed Ct values.

Based on these results, the optimized qPCR mix was defined as 2X PerfeCTa^®^ qPCR FastMix^®^ Low ROX (Cat. 95078-250, Quantabio Beverly, MA, USA), 900 nM H8O26 F1, 300 nM H8O26 R1, and 250 nM H8O26 MGB probe (Table S1). Reactions were completed with 5 µL of extracted DNA for a final volume of 20 µL. This configuration provided the most consistent amplification and extended the assay’s sensitivity to the lowest detectable copy numbers.

### 3.2. Calibrators Study

#### 3.2.1. Comparison Between BCBL-1 and Plasmid Calibrators

Descriptive statistics of Ct values obtained from the qPCR calibration curves generated with both the BCBL-1 cell-derived and the plasmid-based calibrators are summarized in Table S4A,B. Ten-fold serial dilutions ranging from 10^6^ to 1 copy/reaction were tested for both calibrators during 5 days. Amplification was detectable down to a single copy in the BCBL-1-derived material, whereas the plasmid standard yielded consistent amplification only from 10^2^ copies/reaction onward (Table S4).

**Figure 1:**
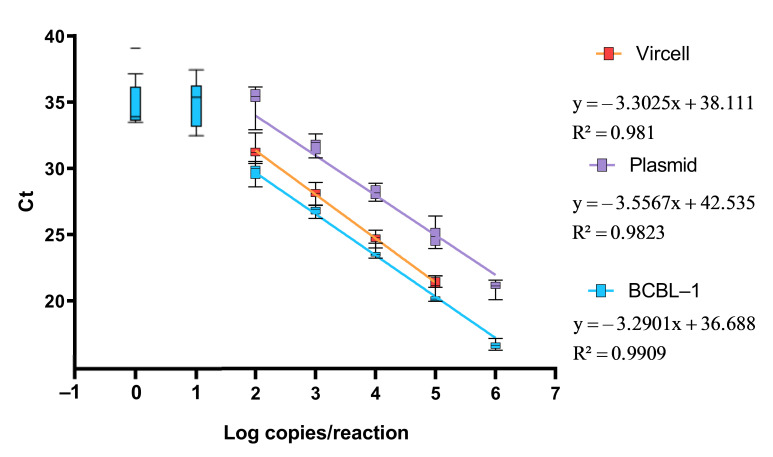
Ct values across cell-derived and plasmid calibrator curves. Box-and-whisker plots showing the distribution of Ct values for each dilution point of the three qPCR calibrators. Boxes represent interquartile ranges (IQR), whiskers the 10th–90th percentiles, and horizontal lines the medians. Lines connecting means illustrate the linear relationship within the validated range (10^6^–10^2^ copies/reaction).

Analysis of the complete 10^6^–1 range for the BCBL-1 curve yielded a slope of −2.919 (E = 120.1%) with R^2^ < 0.92, indicating non-linear behavior at the lowest dilutions. Restricting the analysis to 10^6^–10^1^ copies/reaction improved efficiency to 108.1% (R^2^ > 0.98), while the refined range of 10^6^–10^2^ produced a slope of −3.29 and an efficiency of 104.4%, which was considered optimal [19] (Figure S3). Within this range, both the BCBL-1 and plasmid standards showed strong linearity (R^2^ > 0.98) and comparable amplification performance, with slopes of −3.29 and −3.55, respectively. Below 10^2^ copies/reaction (2 log_10_ copies/reaction), deviations from linearity and loss of exponential amplification were again observed. Figure 1 illustrates the agreement among calibrators.

**Figure 2:**
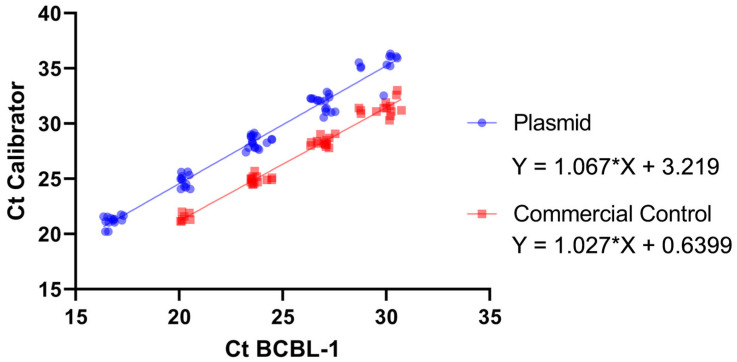
Correlation between BCBL-1 and plasmid- or commercial-based calibrators. Linear regression analysis comparing Ct values of the BCBL-1 calibrator with those from the plasmid (blue) and commercial control (red). Each point represents the mean Ct for a given dilution within the validated dynamic range (10^6^–10^2^ copies/reaction). Dotted lines indicate regression fits with 95% prediction intervals (R^2^ > 0.97).

To further evaluate their equivalence, Ct values from both calibrators were correlated by linear regression within the validated range (10^6^–10^2^ copies/reaction). As shown in Figure 2, the relationship followed the equation *y* = 1.0668*x* + 3.2192 (R^2^ = 0.976). This regression defines a quantitative conversion factor between the plasmid- and cell-based standards, confirming their traceable interconversion and supporting their use as interchangeable reference materials in subsequent analyses.

#### 3.2.2. Comparison of BCBL-1 Lots and Plasmid Performance

Amplification profiles obtained using equivalent amounts of total DNA from two independent BCBL-1 cell batches (050809 and 150111) are shown in Figure S4. Lot 150111 amplified earlier than Lot 050809, suggesting a higher viral genome abundance.

All three calibration curves (two BCBL-1 lots and the plasmid) exhibited strong linearity across the 10^5^–10^2^ copies/reaction range, with slopes between −3.31 and −3.67 (R^2^ > 0.97). Analysis of covariance (ANCOVA) revealed that the interaction between calibrator and log copy number was not significant (*p* = 0.1945), confirming equivalent amplification efficiency between both lots. However, a significant difference was detected in the intercepts, reflecting a shift in baseline Ct values attributable to differences in viral DNA abundance among BCBL-1 lots.

#### 3.2.3. Comparison Between BCBL-1 and the Commercial Control

To further assess the equivalence of the in-house calibrator, Ct values from the BCBL-1 cell-derived standard were correlated with those obtained using the commercial control within the validated dynamic range (10^6^–10^2^ copies/reaction) (Figure 1). Regression analysis showed a linear correlation (R^2^ = 0.974) with the equation *y* = 1.027*x* + 0.64, indicating similar amplification efficiency between both calibrators (Figure 2).

### 3.3. Analytical and Clinical Validation of the qPCR Assay: Linearity, Precision, LOD, and Matrix Effect

#### 3.3.1. Analytical Validation

Linearity was assessed using seven ten-fold dilutions of the BCBL-1 calibrator (10^6^–10^1^ copies/reaction) analyzed over 20 non-consecutive days by two operators in accordance with CLSI EP06-A [16]. The combined dataset demonstrated linear behavior within the 10^6^–10^2^ interval (slope −3.30, intercept 38.59, R^2^ = 0.997; Table S5). Amplification efficiencies obtained from independent runs ranged from 90% to 101%.

Precision was evaluated following CLSI EP05-A2 [15]. Within the validated linear interval (10^6^–10^2^ copies/reaction), Ct variability remained within SD ≤ 0.86 cycles and CV% ≤ 14.2% (Table S5). Below 10^2^ copies/reaction, increased dispersion (CV% > 70%) was observed, defining 10^2^ copies/reaction as the lower limit of quantification.

Operator-specific analyses (Table S6) showed CV% values of 1.7–10.9% across 10^6^–10^2^ copies/reaction, while inter-operator CV% derived from combined data ranged from 2.3% to 14.2%. Independently generated standard curves (Figure S5) demonstrated comparable slopes (−3.49 and −3.41) and coefficients of determination (R^2^ = 0.981 and 0.990).

The 95% limit of detection (LOD_95_%) was estimated using the POD-LOD method and corresponded to 65.68 copies/reaction (95% CI: 46.65–92.68), equivalent to 1.81 log_10_ copies/reaction. Results below this concentration were classified as detected but not quantifiable.

Matrix effect assessment: Across all concentration levels tested (10^6^–10^1^ copies/reaction), matrix-supplemented reactions showed a consistent Ct shift of 1.5–2.5 cycles relative to TE-based reactions. The regression parameters obtained under both conditions remained comparable (BCBL-1 in TE: slope −3.30, R^2^ = 0.997; BCBL-1 + matrix: slope −3.51, R^2^ = 0.985), indicating preservation of curve parallelism within the evaluated range (Figure S6).

Analytical specificity was confirmed by the absence of amplification from HSV-1, HSV-2, VZV, CMV, and EBV.

#### 3.3.2. Clinical Precision Assessment

Clinical precision of qPCR was evaluated using two whole-blood samples representing high and low HHV-8 viral loads (approx. 10^4^ and 10^2^ copies/reaction, respectively). Intra-assay repeatability, intermediate precision, and intra-laboratory precision were calculated from Ct variability across replicates, days, and total measurements

For both viral load levels, repeatability (within run) CV% ranged from 4.4% to 4.6%, whereas intermediate (between day) precision CV% ranged from 2.3% to 4.2%. Intra-laboratory (overall) precision CV% between 4.98% and 6.2%. These results show that Ct variability remained stable across measurement conditions and within the expected range for qPCR-based assays, particularly near the lower viral load concentrations chore increased dispersion is anticipated according to MIQE and EP05-A2 [15,20]. Results are shown in Table 1

### 3.4. Diagnostic Performance Comparison

The clinical validation of the assay was performed on a qualitative basis, following CLSI recommendations for evaluation of diagnostic tests [17,21]. Detection of HHV-8 DNA (positive/negative) by qPCR was compared with conventional endpoint PCR in 121 clinical samples, using the clinical classification described in Section 2.7.2 as the reference standard. The in-house qPCR achieved 100% sensitivity, specificity, PPV, and NPV, whereas endpoint PCR missed two positive cases, resulting in 96.4% sensitivity and 97.1% NPV. McNemar’s test showed no significant difference between the methods (*p* = 0.48). The full contingency analysis is provided in Table S7. These results demonstrate that the in-house qPCR performs at least as accurately as endpoint PCR while adding the advantage of quantitative assessment, with diagnostic metrics surpassing the ≥95% threshold generally recommended for clinical implementation [21,22].

### 3.5. Viral Load Distribution Across Clinical Conditions

Quantitative HHV-8 viral loads (VLs) were obtained for 35 patients diagnosed with epidemic Kaposi’s sarcoma (E-KS), classic KS (C-KS), multicentric Castleman disease (MCD; HIV-positive and HIV-negative), IRIS-associated KS (IRIS-KS), primary effusion lymphoma (PEL), and post-transplant KS (Tx). Viral loads were expressed both as log_10_ copies/mL of whole blood and as log_10_ copies/ng of extracted DNA to assess whether DNA yield influenced inter-group comparisons.

Because the validated reportable range begins at ≥10^2^ copies/reaction, samples yielding Ct values below the linear range were classified as detected but not quantifiable (DNQ). When clinically indicated, DNQ samples were re-extracted and eluted in a lower volume to increase template concentration and enable quantification within the calibrated interval.

VLs expressed as log_10_ copies/mL showed a wide dynamic range with characteristic patterns across conditions:

• Epidemic KS (E-KS, *n* = 15): values ranged from 3.76 to 9.17 log_10_ copies/mL, with a median near 5.5 log_10_; this group included the highest VLs in the cohort (>8.8 log_10_).
• Classic KS (C-KS, *n* = 4): VLs were low and tightly clustered (3.88–4.32 log_10_), consistent with limited systemic involvement.
• MCD HIV-positive (*n* = 7): elevated values (5.66–8.40 log_10_), overlapping the upper E-KS range.
• MCD HIV-negative (*n* = 2): lower VLs (4.47–4.60 log_10_), similar to C-KS.
• IRIS-KS (*n* = 4): consistently high VLs (5.61–7.48 log_10_), overlapping E-KS and MCD HIV+.
• PEL (*n* = 1): 5.41 log_10_ copies/mL, within the range of systemic inflammatory conditions.
• Post-transplant KS (Tx, *n* = 2): moderate VLs (3.78 and 4.08 log_10_), comparable to C-KS.

**Figure 3:**
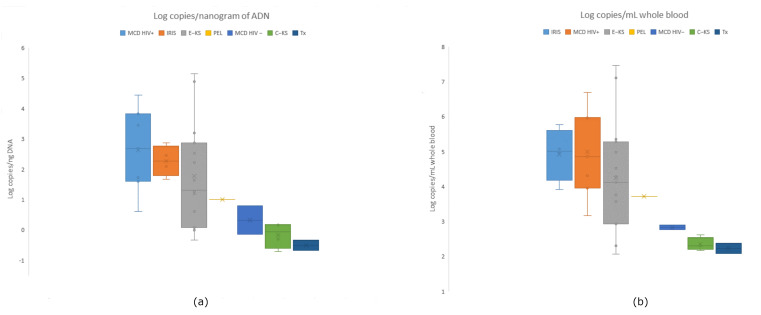
HHV-8 viral load distribution across clinical groups, expressed as log_10_ copies/mL of whole blood and log_10_ copies/ng DNA. Viral loads are shown using two normalization units: (**a**) log_10_ copies/mL of whole blood; (**b**) log_10_ copies/ng of extracted DNA. Because the units differ by a constant scaling factor, normalization does not alter the relative ranking of clinical groups.

These patterns are illustrated in Figure 3, where E-KS, IRIS-KS, and MCD HIV+ show the highest medians and widest IQRs, while C-KS and Tx show the lowest distributions.

#### 3.5.1. Effect of Normalization (log_10_ Copies/ng DNA)

Normalization yielded the same pattern of group differences observed with values expressed per mL of whole blood:

• Higher medians: E-KS, IRIS-KS, MCD HIV+ (≈1.3–3.8 log_10_ copies/ng);
• Lower medians: C-KS, Tx, MCD HIV-negative (≈0 to slightly positive).

These results indicate that variation in DNA yield did not influence inter-group comparisons (Figure 3). Medians by group are summarized in Table 2.

#### 3.5.2. Statistical Analysis of Group Differences

A global Kruskal–Wallis test demonstrated significant VL differences among clinical groups (H = 15.34, *p* = 0.0178). Pairwise post hoc comparisons using Mann–Whitney U tests with Bonferroni correction (adjusted α = 0.0024) identified one statistically significant contrast: C-KS vs. MCD HIV+: VL significantly lower in C-KS (U = 0, *p* = 0.0015). See Table 3.

Other comparisons—such as E-KS vs. IRIS-KS or E-KS vs. MCD HIV+—showed nominal significance (*p* < 0.05) but did not remain significant after correction, reflecting overlapping distributions in high-viremia categories.

When required, viral loads originally expressed as log_10_ copies per ng of extracted DNA were converted to log_10_ copies per 10^6^ cells by applying a constant scaling factor. Assuming an average diploid human genome mass of 6.6 pg per cell, 1 ng of DNA corresponds to approximately 151 cells; therefore, 10^6^ cells contain ~6606 ng of DNA. The transformation to copies per 10^6^ cells is thus obtained by adding log_10_ (6606) = 3.82 to the values expressed as log_10_ copies/ng. Because this conversion involves a fixed linear shift, it does not alter the relative distribution of viral loads across samples

## 4. Discussion

In this study, we developed and validated a real-time PCR assay for the quantification of HHV-8 DNA in whole blood, following a structured workflow that encompassed assay design, optimization, analytical validation, calibrator evaluation, and clinical application. The assay was conceived to overcome the limitations of the nested PCR routinely used in our laboratory, which, although highly sensitive, does not allow viral load quantification or longitudinal monitoring [23].

The design phase included the evaluation of two TaqMan probe systems and multiple primer–probe combinations informed by the full spectrum of ORF26 genotypes described worldwide, together with locally circulating variants previously identified in our setting. This comprehensive approach ensured compatibility with global HHV-8 sequence diversity and reduced the likelihood of genotype-dependent differences in amplification performance.

The use of plasmid and cell-derived standards enabled a thorough assessment of assay performance in line with MIQE recommendations for transparent validation of qPCR assays [20]. Within the linear range of the calibration curve, the standard deviation of replicate Ct measurements consistently remained below 1.66 cycles (≈0.5 log), fulfilling the precision limits defined by CLSI EP05 for quantitative assays [15]. Across most dilution points, plasmid and BCBL-1 calibrators showed comparable variability, although the cell-derived material more closely reflected the behavior of clinical specimens, as also noted in other virological qPCR studies [22,24].

Despite minor deviation at the lowest dilution point, the overall correlation between calibrators was strong (R^2^ > 0.98), and a correction factor was established to allow reliable interconversion.

For each new batch of BCBL-1 cells, a fresh calibration curve is generated and recalibrated against the plasmid standard to maintain traceability and ensure continuity between batches. The use of a cell-derived calibrator offers a key practical advantage: because it undergoes the same DNA extraction and amplification workflow as clinical specimens, it more accurately reflects routine analytical conditions. Plasmid standards, although stable and precise, must be handled in a physically separate amplification area to minimize contamination risk, which disrupts workflow continuity. For this reason, BCBL-1 material is used as the primary working calibrator, whereas the plasmid standard serves as a fixed reference for periodic verification.

Comparative analysis of plasmid-based, commercial, and cell-derived calibrators demonstrated that the in-house BCBL-1 standard supports reliable HHV-8 quantification across the validated dynamic range of 10^6^–10^2^ copies/reaction. Linearity and efficiency (E = 104–108%, R^2^ > 0.98) remained stable regardless of the calibrator source, and Ct distributions did not differ significantly at equivalent dilution points. These results confirm that the BCBL-1 calibrator maintains quantitative equivalence with external standards while offering the biological realism of a true cell-based matrix and reducing dependence on commercial reagents.

Although direct inter-laboratory evaluation was not performed, the calibration framework established here enables reproducible conversion between cell-derived, plasmid, and commercial standards, providing traceable alignment with externally validated materials. The strong linear correlation observed (R^2^ = 0.97–0.98) indicates quantitative equivalence across calibrators and supports the use of this system as a reliable bridge toward broader comparability.

Evaluation of two independent BCBL-1 lots showed consistent amplification efficiencies (*p* = 0.19), indicating stable reaction kinetics across batches. However, differences in intercepts reflected variation in viral genome abundance, likely attributable to fluctuations in lytic activity or the proportion of infected cells in each culture. This underscores the need to verify lot-to-lot equivalence—using regression or ANCOVA—before adopting a new batch as a reference. Although reaction efficiency remained constant, each cell-derived batch required its own adjustment relative to the plasmid standard to ensure accurate quantitation.

This approach aligns with current efforts in molecular diagnostics to achieve standardization through traceability to certified external controls, rather than through isolated exchanges among individual laboratories. By integrating a commercial HHV-8 DNA control (Vircell) as an external reference, this study provides a scalable and reproducible pathway toward future inter-laboratory harmonization.

Overall, these findings demonstrate that the BCBL-1 calibrator functions as a consistent, renewable, and biologically relevant standard for HHV-8 quantification, provided that each new batch is cross-validated against the plasmid reference prior to implementation.

The in-house qPCR assay was developed to overcome the limitations of the nested PCR routinely used in our laboratory, which, although sensitive, did not allow for quantification or monitoring of viremia [10]. The final protocol, targeting the ORF26 region and using the FAM–MGB probe, achieved acceptable analytical performance, with efficiencies of 90–101% and R^2^ values > 0.99, in accordance with MIQE requirements for reporting and validating qPCR performance parameters [20]. The limit of detection was estimated at 65.68 copies/reaction. Reproducibility was high, with intra- and inter-assay coefficients of variation ≤ 7%. Although MIQE does not define numerical acceptance criteria for precision, it emphasizes transparent reporting of replicate variability. In contrast, CLSI EP05-A2 provides quantitative benchmarks, allowing up to 15% CV for intermediate precision and up to 20% at the lower limit of quantification [15]. Our values fall well within these ranges and compare favorably with published qPCR assays in virology, where intra-assay CVs typically range from 2–5% and inter-assay variability remains below 10% [25]. In all runs, Ct variation remained ≤1.5 cycles, further supporting the robustness of the assay for routine diagnostic use

Whole blood was selected as the matrix because it captures both the cell-associated and the cell-free fractions of HHV-8. Despite HHV-8’s cell-associated nature [26], episodes of lytic replication may lead to high circulating DNA levels even when PBMC counts are low [27]. This broader representation of viral forms is advantageous not only for clinical monitoring—particularly in immunocompromised patients—but also for research purposes, as frozen whole-blood specimens from blood banks can be used for retrospective analyses or to explore the potential for transfusion-related transmission. In addition, whole blood is easy to collect, store, and process, making it a practical and accessible specimen for routine diagnostics.

Comparisons of reported HHV-8 VLs in different studies are often hampered by the lack of standardization in units. Some express results as copies or log copies per ml of plasma [28] others per microgram of DNA [29]; or per 10^6^ cells [30,31].

Although viral loads can be expressed either as copies/ng of DNA or as copies per 10^6^ cells, these units are linearly related by a constant factor. Therefore, converting between them does not affect the relative distribution of viral loads across samples.

Our study contributes to this discussion by using a validated calibration approach that facilitates reproducibility and comparison. A recent report comparing two commercial kits for HHV-8 quantification showed viral load values in whole blood that were comparable to those found in our study, log copies/mL [32].

We also explored potential associations between viral load and clinical or demographic variables. Although no significant differences were found by sex or clinical form, an inverse correlation with age was observed: younger patients—particularly those with E-KS or MCD HIV+—tended to have higher viral loads. In contrast, classic KS (C-KS), more frequent in older individuals, showed consistently low values.

These findings are supported by previous studies showing that high HHV-8 DNAemia is associated with active or inflammatory disease forms such as epidemic KS, IRIS-KS, and HIV-positive MCD [3,27,31], while classic and transplant-associated KS cases generally present with low viremia consistent with their indolent course [29,30]. Furthermore, younger, HIV-positive patients tend to exhibit higher HHV-8 DNAemia than older, HIV-negative cases [4,29]. Longitudinal data also show that viral load declines with effective therapy or clinical improvement [26,28,32].

In contrast, classic and transplant-associated KS cases consistently exhibited low-level viremia, aligning with their typically indolent clinical course [29,30,31]. Notably, two E-KS samples also showed unexpectedly low VLs. Upon review, one belonged to a patient recently transfused, and the other had been transported from a distant region and improperly stored at 4 °C for two months—circumstances likely contributing to DNA degradation and underestimation of viral burden.

While only the C-KS vs. MCD HIV+ comparison retained statistical significance after Bonferroni correction (*p* = 0.0015), other contrasts—particularly involving E-KS, IRIS-KS, and MCD HIV+—displayed consistent biological patterns. Together, these findings highlight the clinical relevance of HHV-8 quantification, particularly in identifying patients with active or inflammatory disease, and support its potential role in guiding patient stratification and monitoring therapeutic response [3,27].

Viral load differences across clinical groups provided meaningful insight into HHV-8 disease activity and immune status. The highest VLs (>5 log copies/mL) were observed in two E-KS patients with florid, poorly controlled disease: one was resistant to liposomal doxorubicin and switched to paclitaxel, while the other failed to suppress HIV replication despite one year of cART. Similarly, high VLs were detected in MCD HIV+ patients and four individuals with IRIS-KS. Among the latter, two presented with bone marrow aplasia and intense systemic inflammation; a third later developed KICS and concomitant MCD. These findings underscore the assay’s potential to monitor HHV-8–driven inflammatory complications in the context of immune reconstitution [1,31].

Notably, longitudinal follow-up in selected cases revealed that clinical improvement following therapy coincided with viral load decline [26,28,32], reinforcing the clinical utility of this qPCR assay for disease monitoring.

Nevertheless, the relatively small number of samples per group may have limited the power to detect additional associations. See Figure S7 Future multicentric studies with larger cohorts could clarify these trends.

Conclusion: We established a quantitative real-time PCR assay for HHV-8 in whole blood supported by a calibration strategy that integrates plasmid and cell-derived standards. Comparative analysis demonstrated a stable quantitative relationship among calibrators and confirmed that the BCBL-1–derived material functions as a reliable working standard, provided that each lot is cross-validated against a fixed plasmid reference. Analytical validation met CLSI performance requirements for linearity, efficiency, precision, and detection limits, and clinical evaluation showed complete agreement with a composite diagnostic standard. The assay enabled characterization of viral load patterns across HHV-8–associated diseases and supported longitudinal monitoring.

Beyond analytical validation, this study demonstrates the translational potential of HHV-8 DNA quantification for patient care. Although statistical significance was achieved primarily for the comparison between C-KS and MCD HIV+, consistent biological patterns across other clinical subgroups (E-KS, IRIS-KS, and MCD HIV+) underscore the clinical relevance of viral load as an indicator of disease activity and inflammation. Moreover, longitudinal follow-up showed that viral load decline paralleled clinical improvement, highlighting the assay’s applicability for monitoring therapeutic response.

The methodological framework described here was designed to be transferable beyond our local setting. By integrating inter-convertible plasmid, cell-derived, and commercial calibrators, and by demonstrating equivalence among reporting units, this study establishes a reproducible foundation for cross-laboratory comparability in HHV-8 quantification. Although developed within a national reference center, the approach is adaptable to other diagnostic laboratories using compatible qPCR platforms and could serve as a reference model for multicentric or international harmonization initiatives aimed at standardizing HHV-8 viral load measurement.

## Figures and Tables

**Table 1:** Precision parameters obtained from high and low viral-load whole-blood controls.

Parameter	High Control (Ct ≈ 22.4)	Low Control (Ct ≈ 30.4)
Intra-assay repeatability (SD)	1.02	1.34
Intermediate precision (SD)	0.94	0.71
Intralaboratory precision (SD)	1.38	1.51
Intra-assay repeatability (CV%)	4.6%	4.4%
Intermediate precision (CV%)	4.2%	2.3%
Intralaboratory precision (CV%)	6.2%	4.98%

**Table 2:** Median HHV-8 viral loads (log_10_) by clinical group.

Clinical Group	Median (log_10_ Copies/ng DNA)	Median (log_10_ Copies/mL Whole Blood)	Δ (mL − ng)	Rank (ng)	Rank (mL)
IRIS	2.48	5.02	+2.54	1	1
MCD HIV+	2.69	4.86	+2.17	2	2
E-KS	1.64	4.12	+2.48	3	3
PEL	1.01	3.72	+2.71	4	4
MCD HIV−	0.33	2.84	+2.51	5	5
C-KS	0.18	2.32	+2.14	6	6
Tx-KS	−0.50	2.23	+2.73	7	7

Median HHV-8 viral loads (log_10_) by clinical group, expressed per ng DNA and per mL of whole blood. Δ represents the difference between normalization units (mL − ng). Ranks correspond to the order of medians within each unit.

**Table 3:** Post hoc pairwise comparison of HHV-8 viral load between clinical groups.

Comparison	Mann–Whitney U	*p* Value	Bonferroni-Adjusted α	Significance
C-KS vs. MCD HIV+	0	0.0015	0.0024	Significant
C-KS vs. E-KS	—	0.028	0.0024	ns
E-KS vs. IRIS-KS	—	0.307	0.0024	ns
E-KS vs. MCD HIV+	—	0.267	0.0024	ns

Footnote: Pairwise comparisons were performed using the Mann–Whitney U test following a significant Kruskal–Wallis test. Bonferroni correction was applied for multiple testing (adjusted α = 0.0024). Only the comparison between classic Kaposi’s sarcoma (C-KS) and HIV-associated multicentric Castleman disease (MCD HIV+) remained statistically significant; ns: not significant

## Data Availability

The datasets generated and/or analyzed during the current study are available from the corresponding author on reasonable request.

## Supplementary Materials

The following supporting information can be downloaded at: https://www.mdpi.com/article/10.3390/v18050578/s1, Figure S1: Primers and probes position in ORF 26 alignment; Figure S2: Graphical analysis of FAM-TAMRA and FAM –MGB systems; Figure S3: Amplification curves of the BCBL-1-derived calibrator; Figure S4: Cts across different cell lots and plasmid; Figure S5: Operator-specific standard curves for the HHV-8 qPCR assay; Figure S6: Whole blood Matrix effect on calibration curve; Figure S7: Viral load follow up; Table S1: Primer and probe sets of HHV-8 ORF 26 fragment; Table S2: Ct Results for 10^3^ Copies Using Primer-Probe Combinations for FAM-TAMRA and FAM-MGB; Table S3: Mean Ct Values (±SD) for Each Log Concentration in a six points calibration Curve; Table S4: Descriptive Statistics of Ct Values for qPCR Calibrators; Table S5: Summary statistics of the combined dataset used for the analytical validation of the HHV-8 qPCR assay; Table S6: Descriptive statistics and precision parameters for each operator across dilution points; Table S7: Diagnostic performance comparison among PCRs; Table S8: Demographic characteristics and HHV-8 laboratory results. 

## Abbreviations

INEI	National Institute of Infectious Diseases
ANLIS	National Administration of Laboratories and Institutes of Health
VL	Viral Load
HHV-8	*Human herpesvirus*-8
KSHV	Kaposi’s sarcoma-associated herpesvirus
cART	combined antiretroviral therapy
PEL	primary effusion lymphoma
MCD	Multicentric Castleman’s disease
KS	Kaposi’s sarcoma
TE	Tris-EDTA
