# Supplementary material for: Validated Quantification of HHV-8 DNA Using Inter-Convertible Plasmid and Cell-Derived Calibrators: Optimization of a Whole-Blood qPCR Assay
Source: Viruses. 2026 May 21;18(5):578. doi: 10.3390/v18050578 (PMC13211678; doi:10.3390/v18050578)

Supplementary Figures- Validated Quantification of HHV-8 DNA Using Inter-Convertible Plasmid and Cell-Derived Calibrators: Optimization of a Whole-Blood qPCR Assay

Figure S1. Primers and probes position in ORF 26 alignment

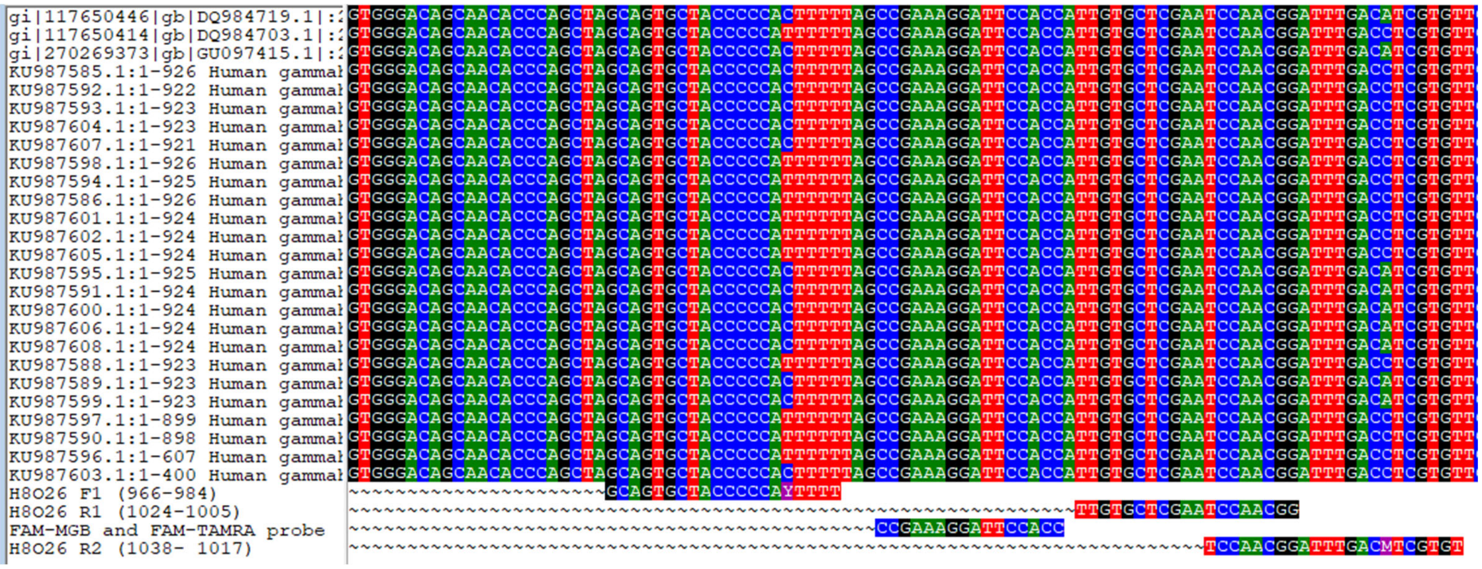

Figure S2 Graphical analysis of FAM-TAMRA and FAM –MGB systems

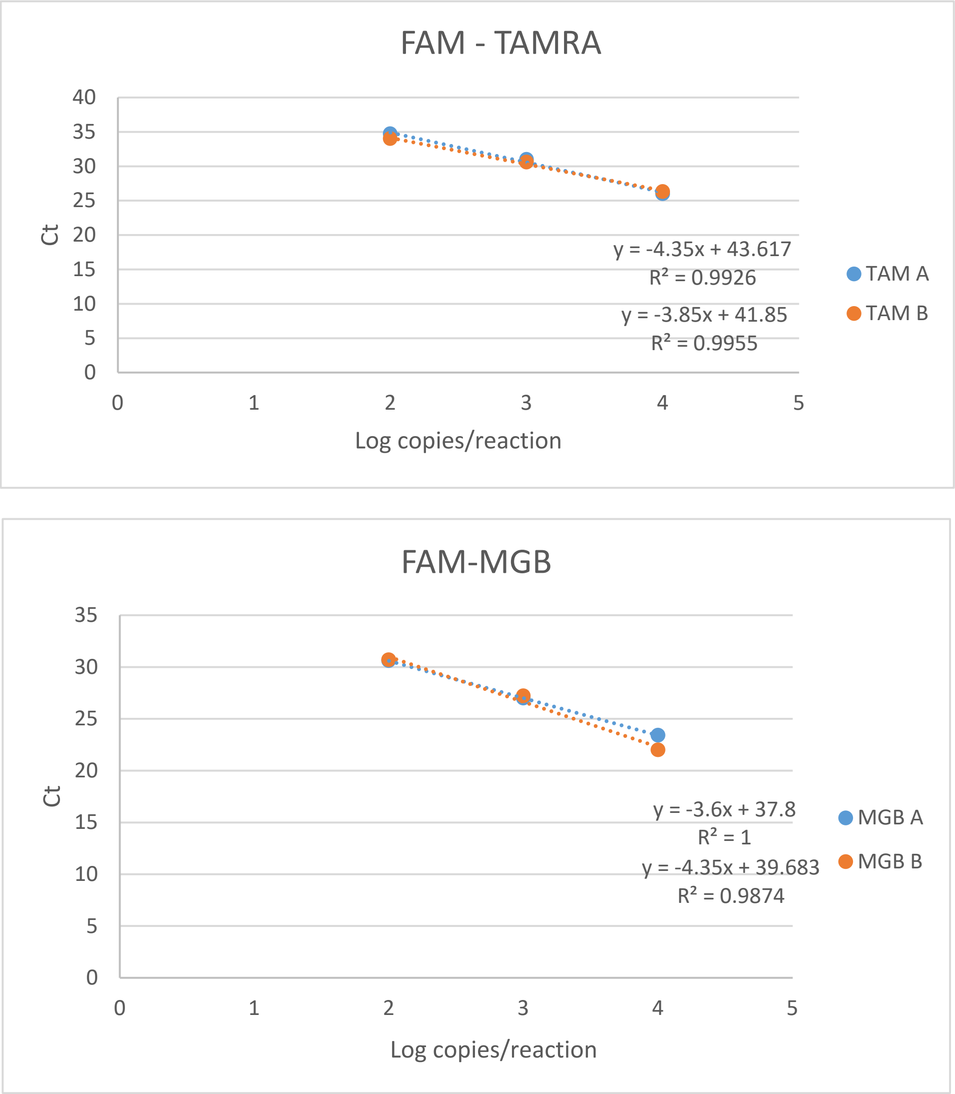

(a) Graphical analysis of the efficiency of the FAM-TAMRA system, showing its linear equations with slopes of 4.35 and 3.85 for combinations A and B, respectively;(b) FAM-MGB system, presenting slope values of 3.6 and 4.35 for combinations A and B respectively.

Figure S3. Amplification curves of the BCBL-1-derived calibrator.

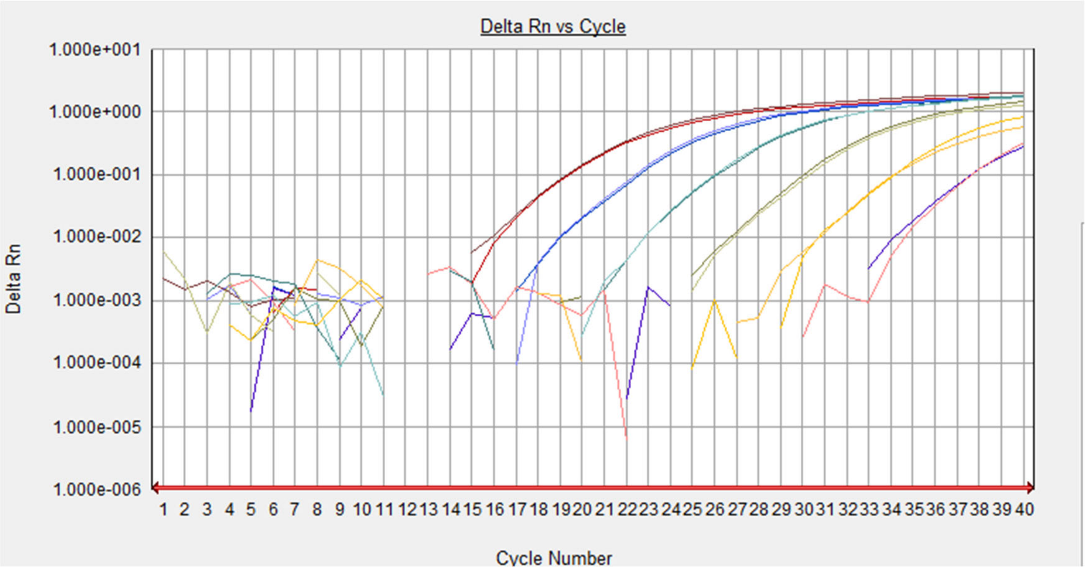

Fluorescence amplification plots (ΔRn vs. cycle number) showing six ten-fold serial dilutions (10^6–10^1 copies/reaction) of the BCBL-1-derived calibrator. Curves illustrate the typical amplification behaviour across the range used for the construction of the standard curve.

Figure S4. Cts across different cell lots and plasmid

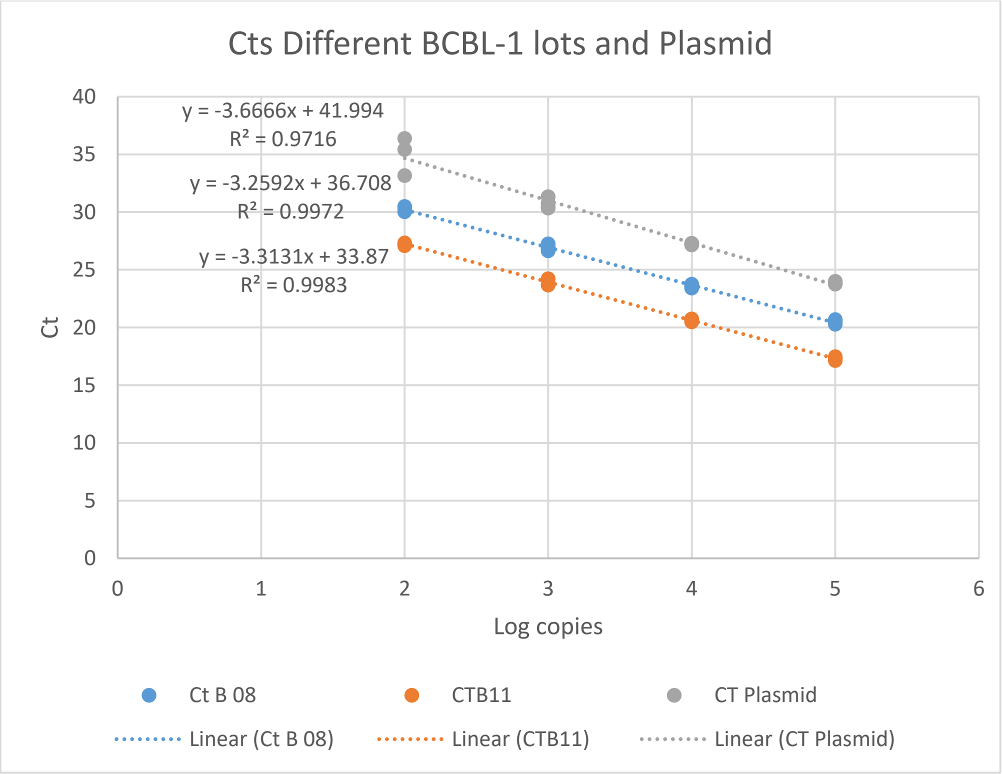

Each point represents the mean ± SD of triplicate Ct values per dilution. All curves showed comparable slopes ($p = 0.19$) but differed in intercepts, reflecting variations in viral genome content between BCBL-1 lots.

Figure S5. Operator-specific standard curves for the HHV-8 qPCR assay.

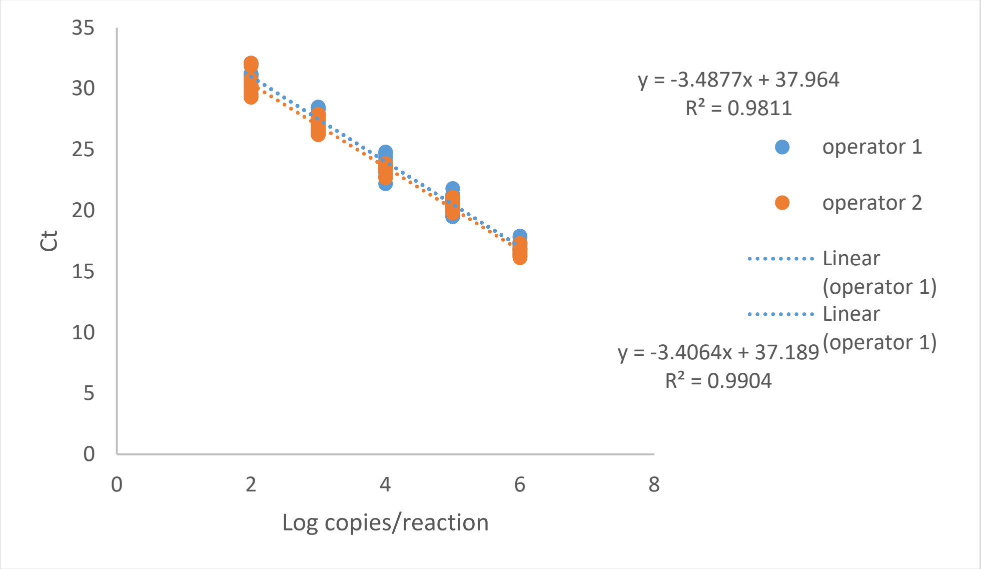

Comparison of calibration curves independently generated by Operator 1 and Operator 2 using BCBL-1 cell-derived DNA dilutions (10^6–10^2 copies/reaction). Regression parameters were $y = -3.49x + 37.96$ ($R^2 = 0.981$) and $y = -3.41x + 37.19$ ($R^2 = 0.990$), corresponding to amplification efficiencies of 93.6 % and 96.6 %, respectively. The overlapping slopes and R^2 values indicate comparable linearity and amplification efficiency between operators.

Figure S6. Whole blood Matrix effect on calibration curve

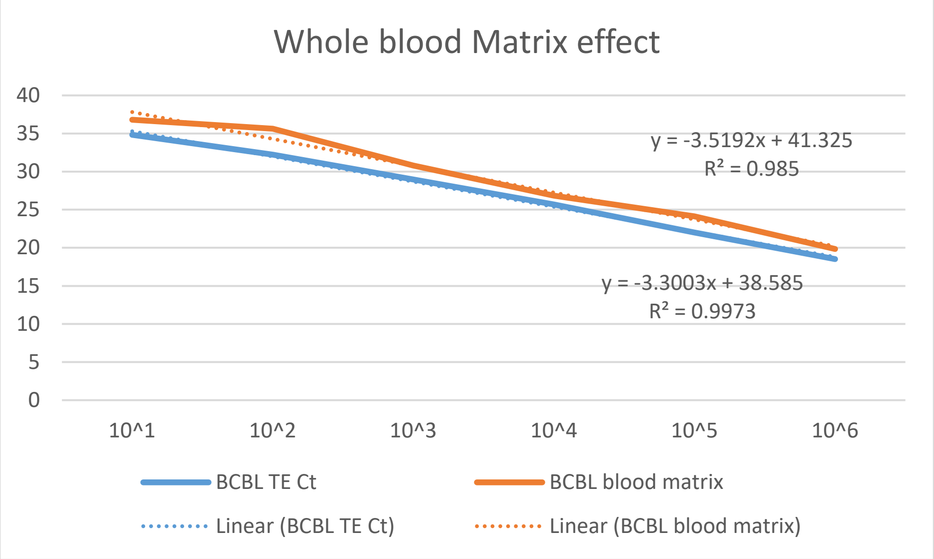

Figure S7. Viral load follow up

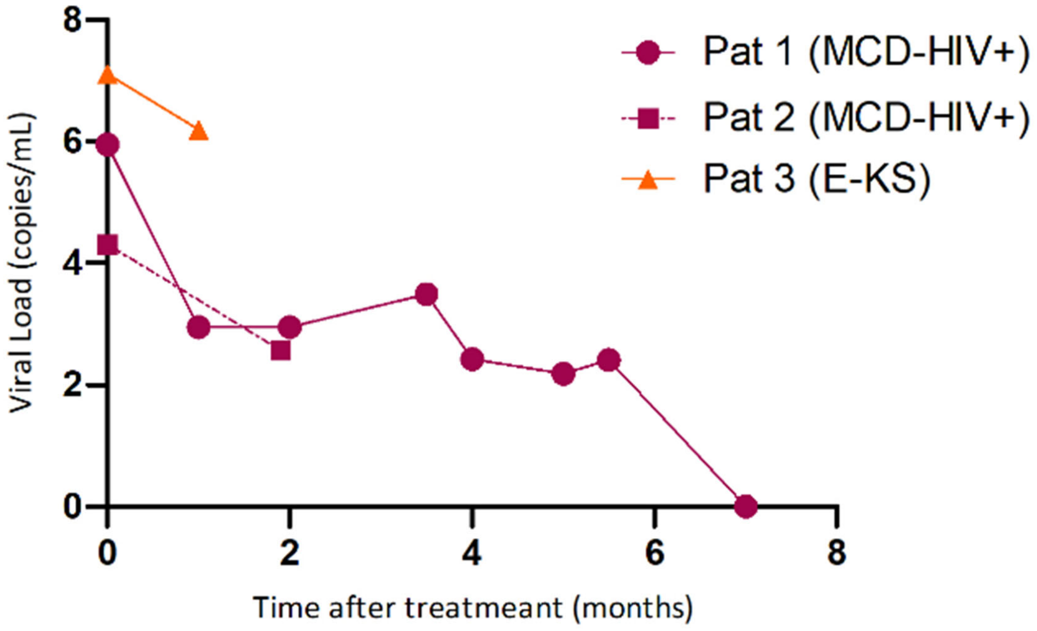

Supplement: Supplementary file 1 [file viruses-18-00578-s001.zip › viruses-4068247-supplementary Figures.pdf]
