# Supplementary material for: Validated Quantification of HHV-8 DNA Using Inter-Convertible Plasmid and Cell-Derived Calibrators: Optimization of a Whole-Blood qPCR Assay
Source: Viruses. 2026 May 21;18(5):578. doi: 10.3390/v18050578 (PMC13211678; doi:10.3390/v18050578)
Supplement: Supplementary file 1 [file viruses-18-00578-s001.zip › viruses-4068247-supplementary Tables.pdf]

Supplementary Tables - Validated Quantification of HHV-8 DNA Using Inter-Convertible Plasmid and Cell-Derived Calibrators: Optimization of a Whole-Blood qPCR Assay

Table S1. Primer and probe sets of HHV-8 ORF 26 fragment

Set	Name Direction nt Source	Sequences **5´3´** - ***Modifications***
TAQMAN MGB-NFQ	H8O26 F1 (966-984)	GCAGTGCTACCCCCA***C/T***TTT -C/T **nt 981**
	H8O26 R1 (1024-1005)	H8O26 F1 (966-984)
	H8O26 MGB probe (989-1004)	***FAM-***CCGAAAGGATTCCACC-***NFQ***
TAQMAN TAMRA	H8O26 F1 (966-984)	GCAGTGCTACCCCCA***C/T***TTT -C/T **nt 981**
	H8O26 R2 (1038- 1017)	ACACGA***G/T***GTCAAATCCGTTGGA G/T nt 1032
	H8O26 TAMRA Probe (989-1004)	**FAM-**CCGAAAGGATTCCACC-**TAMRA**

Note: F: forward, R: Reverse, nt: (nucleotide position) MGB: Minor groove binding. NFQ: Non fluorescent quencher. FAM: 5(6)-Carboxyfluorescein; TAMRA: 5-Carboxytetramethylrhodamine

Table S2. Ct Results for 10^3 Copies Using Primer-Probe Combinations for FAM-TAMRA and FAM-MGB

Probe-F/R (nM)	FAM-TAMRA Ct (Mean ± SD)	FAM-MGB Ct (Mean ± SD)
**125-900/900**	31.93 ± 0.014	**30.00 ± 0.014 (B)**
**125-900/300**	33.14 ± 0.085	**31.09 ± 0.014 (A)**
**125-300/900**	**31.44 ± 0.014 (B)**	31.52 ± 0.233
125-300/300	33.84 ± 0.658	32.35 ± 0.474
250-900/900	31.98 ± 0.403	31.39 ± 0.354
250-900/300	32.41 ± 0.064	31.01 ± 0.679
**250-300/900**	**30.19 ± 0.028 (A)**	34.92 ± 0.792
250-300/300	32.10 ± 1.223	32.45 ± 1.103

Note: F: forward primer; R: reverse primer; nM: concentration for R or F. Details of probes and primers are depicted in table 1S. In bold: concentrations chosen for further study named A and B in each system

Table S3. Mean Ct Values (± SD) for Each Log Concentration in a six points calibration Curve

**Log copies**	**FAM-TAMRA (Media ± SD)**	**FAM-MGB (Media ± SD)**
6	22,48 ± 0,15	19,4 ± 0,16
5	26,09 ± 0,06	22,43 ± 0,07
4	30,31 ± 0,51	26,66 ± 0,28
3	33,65 ± 0,03	30,05 ± 0,09
2	38,13 ± 0,11	33,32 ± 0,00
1	-	36,4 ± 0,00

Note: Standard curve formulas and efficiency values were: FAM-TAMRA: $Y = 3.886X + 45.67$, $R^2 = 0.998$; FAM-MGB: $Y = 3.458X + 40.1$, $R^2 = 0.998$

Table S4. Descriptive Statistics of Ct Values for qPCR Calibrators

Log copies	Mean Ct	95% CI lower	95% CI Upper	Median Ct	SD	Min	Max	Range	IQR
									A. BCBL-1 Cell-Derived Calibrator
0	33.61	32.16	35.06	32.97	1.89	31.90	37.35	5.45	3.13
1	32.74	31.95	33.53	32.39	1.36	30.95	35.18	4.23	2.10
2	29.88	29.48	30.27	30.09	0.68	28.68	30.74	2.06	0.70
3	26.91	26.74	27.08	27.01	0.36	26.23	27.54	1.31	0.52
4	23.71	23.55	23.87	23.63	0.34	23.23	24.49	1.25	0.27
5	20.25	20.15	20.36	20.20	0.16	20.08	20.54	0.46	0.28
6	16.77	16.60	16.95	16.77	0.31	16.35	17.31	0.96	0.41
									B. Plasmid-Based Calibrator
0	ND								
1	ND								
2	35.39	34.69	36.09	35.54	1.05	32.54	36.31	3.77	0.90
3	31.84	31.49	32.20	32.09	0.67	30.55	32.86	2.30	1.00
4	28.34	28.08	28.61	28.32	0.53	27.41	29.17	1.76	1.01
5	24.82	24.49	25.16	24.86	0.55	24.07	25.63	1.56	0.98
6	21.13	20.84	21.42	21.25	0.52	20.18	21.75	1.57	0.50
									C. Commercial Control
2	31.41	31.01	31.82	31.30	0.71	30.30	33.00	2.70	0.57
3	28.33	28.11	28.55	28.25	0.39	27.80	29.07	1.27	0.45
4	28.33	28.11	28.55	28.25	0.39	27.80	29.07	1.27	0.45
5	21.49	21.16	21.81	21.30	0.35	21.15	22.00	0.85	0.67

Note: Ct = cycle threshold; SD = standard deviation; IQR = inter-quartile range. Data derive from three replicates per dilution across five independent runs. ND: not detected

Table S5 Summary statistics of the combined dataset used for the analytical validation of the HHV-8 qPCR assay.

BCBL-1 calibration curve

Log (Copies/reaction)	Mean Ct	95%CI lower	95%CI upper	Median Ct	SD	Min	Max	Range	IQR	CV%
0	34,66	33,60	35,73	34,94	1,99	31,90	37,40	5,50	3,67	71,8
1	33,79	33,20	34,38	34,11	1,78	30,95	37,63	6,68	3,09	48,7
2	30,67	30,48	30,99	30,55	0,86	29,29	32,10	2,81	1,17	12,8
3	27,16	26,97	27,34	27,12	0,58	26,23	28,50	2,27	0,78	5,6
4	23,57	23,40	23,73	23,61	0,49	22,20	24,80	2,60	0,25	3,6
5	20,42	20,25	20,59	20,38	0,50	19,49	21,80	2,31	0,59	3,0
6	16,89	16,73	17,06	16,86	0,46	16,13	17,90	1,77	0,74	2,2

Table S6. Descriptive statistics and precision parameters for each operator across dilution points.

B. Operator 1

Log Copies/reaction	Mean Ct	95%CI lower	95%CI upper	Median Ct	SD	Min	Max	Range	IQR	CV%
0	37,00	33,19	40,81	37,00	0,42	36,70	37,30	0,60	1,53	96,42
1	35,01	34,42	35,61	35,09	1,23	32,50	37,63	5,13	1,43	50,72
2	31,33	30,88	31,78	31,20	0,67	29,87	32,10	2,23	0,95	10,95
3	27,35	26,89	27,82	27,34	0,77	26,24	28,50	2,26	1,42	7,67
4	23,71	23,32	24,10	23,67	0,67	22,20	24,80	2,60	0,67	4,89
5	20,49	20,19	20,79	20,49	0,63	19,49	21,80	2,31	0,85	3,70
6	17,28	17,07	17,48	17,30	0,34	16,70	17,90	1,20	0,43	1,69

A. Operator 2

Log Copies/reaction	Mean Ct	95%CI lower	95%CI Upper	Median Ct	SD	Min	Max	Range	IQR	CV%
0	34,33	33,23	35,43	34,73	1,90	31,90	37,40	5,50	2,82	61,12
1	32,50	31,87	33,14	32,05	1,28	30,95	35,18	4,23	1,95	25,96
2	30,29	29,94	30,65	30,19	0,74	29,29	32,10	2,81	0,62	10,28
3	27,06	26,88	27,23	27,10	0,44	26,23	27,90	1,67	0,62	4,22
4	23,48	23,34	23,63	23,56	0,33	22,70	23,87	1,17	0,26	2,40
5	20,34	20,18	20,50	20,29	0,33	19,80	21,10	1,30	0,37	1,91
6	16,64	16,48	16,81	16,59	0,35	16,13	17,31	1,18	0,46	1,68

Descriptive parameters (mean Ct, SD, and CV%) calculated from 20 independent runs performed by two operators using the BCBL-1 calibrator across seven ten-fold dilutions (10^6–10^1 copies/reaction). The dataset summarizes the mean Ct variability, confirming precision and linearity within the 10^6–10^2 copies/reaction range used to define the assay's reportable interval.

Table S7. Diagnostic performance comparison among PCRs

	Endpoint PCR Positive	*Endpoint PCR Negative*	*Total*	*Diagnostic metric*	*qPCR (%)*	*End point PCR (%)*
qPCR Positive	53	2	55	Sensitivity	100	96.4
qPCR Negative	0	66	66	Specificity	100	100
Total	53	68	121	VPP	100	100
Diagnostic metric				VPN	100	97.1

Note: Values reflect diagnostic concordance between qPCR and endpoint PCR assays using a sample cohort (n = 121), tested in replicates across multiple runs.

Table S8. Demographic characteristics and HHV-8 laboratory results.

ID	Viral Load (Log10 copies/mL)	Age	Sex	nested PCR						IFA (Serum)
				BAL	Biopsy	CSF	Lymphocytes	Saliva	Whole Blood	
C-KS-1	2.62	48	M	NA	NA	NA	NA	NA	+	+
C-KS-2	2.32	77	M	NA	NA	NA	+	+	+	+
C-KS-4	2.30	86	F	+	NA	NA	-	+	+	+
C-KS-5	2.18	49	M	NA	NA	NA	-	+	+	+
E-KS-1	2.06	33	M	NA	NA	NA	NA	NA	+	+
E-KS-10	2.93	73	M	NA	+	NA	+	+	+	+
E-KS-12	3.58	29	M	NA	NA	+	NA	NA	+	NT
E-KS-13	4.12	19	M	NA	+	NA	+	-	+	+
E-KS-15	4.29	38	M	NA	NA	NA	+	NA	+	NT
E-KS-16	4.53	57	M	-	NA	NA	+	NA	+	+
E-KS-17	4.98	29	M	NA	NA	NA	NA	NA	+	+
E-KS-18	5.29	41	M	NA	+	NA	+	+	+	NT
E-KS-19	7.11	29	M	NA	NA	NA	NA	-	+	NT
E-KS-21	7.47	50	M	NA	+	NA	NA	NA	+	NT
E-KS-4	3.57	42	F	-	+	NA	NA	+	+	NT
E-KS-5	5.36	45	M	NA	+	NA	+	+	+	+
E-KS-6	3.76	33	M	-	NA	NA	+	-	+	NT
E-KS-8	2.30	31	M	NA	NA	NA	-	-	+	NT
E-KS-9	2.33	38	M	NA	+	NA	-	NA	+	NT
MCD-1 HIV+	3.97	55	M	NA	+	NA	+	NA	+	+
MCD-2 HIV+	6.70	37	M	NA	+	NA	NA	NA	+	+
MCD-3 HIV+	4.86	22	M	NA	NA	NA	+	+	+	NT
MCD-4 HIV+	5.98	38	M	NA	+	NA	+	+	+	NT
MCD-5 HIV+	5.97	40	F	NA	NA	NA	+	+	+	+
MCD-6 HIV+	4.31	40	M	NA	NA	NA	+	+	+	+

MCD-7 HIV+	3.17	55	M	NA	NA	NA	+	NA	+	+
MCD-8 [A]HIV-	2.77	62	F	NA	-	NA	NA	-	-	+
MCD-9 HIV-	2.90	39	F	NA	+	NA	-	-	+	NT
PEL-1	3.72	66	M	NA	+	NA	NA	NA	+	NT
IRIS-1	5.08	38	M	NA	+	NA	+	+	+	+
IRIS-2	5.78	32	M	NA	+	NA	NA	NA	+	+
IRIS-3	4.96	22	M	NA	NA	NA	+	+	+	NT
IRIS-4	3.91	35	M	NA	+	NA	+	+	+	+
TX-1[B]	2.38	6	M	NA	-	NA	NA	+	-	NT
TX-2	2.08	29	F	NA	NA	NA	NA	+	+	+

Note. Age is expressed in years. Qualitative results: **(+)**, positive; **(-)**, negative; **NA**, not available; **NT**, not tested. **Abbreviations: BAL**, bronchoalveolar lavage; **CSF**, cerebrospinal fluid; **IFA**, immunofluorescence assay.

Detailed discordant whole blood cases: nested PCR negative/ qPCR positive

Patient ID	Sample	Disease	Nested PCR	qPCR	Serology (IFA)	Comments / Notes
**MCD-08[A]**	Lymph node biopsy	Castleman disease	Indeterminate (reported as negative)	Positive	Positive	Weak end-point PCR signal; reported as negative but positive by qPCR, whole blood negative by nested PCR but positive by qPCR.
**TX-1[B]**	Hepatic biopsy / Whole blood	Post-transplant (6-year-old)	Negative (10 µL input)	Positive	NT	Hepatic biopsy positive by nested PCR (10 µL extract); whole blood negative by nested PCR but positive by qPCR.
